# Effects of high-intensity interval training on lean mass, strength, and power of the lower limbs in healthy old and young people

**DOI:** 10.3389/fphys.2023.1223069

**Published:** 2023-09-27

**Authors:** Cristian Caparrós-Manosalva, Nicolás Garrido-Muñoz, Bastián Alvear-Constanzo, Sofía Sanzana-Laurié, Macarena Artigas-Arias, Andrea Alegría-Molina, Nicolás Vidal-Seguel, Jessica Espinoza-Araneda, Nolberto Huard, Aline Souza Pagnussat, Jorge Sapunar, Luis A. Salazar, Gabriel Nasri Marzuca-Nassr

**Affiliations:** ^1^ Department of Human Movement Sciences, Faculty of Health Sciences, University of Talca, Talca, Chile; ^2^ Rehabilitation Sciences Graduate Program, Universidade Federal de Ciências da Saúde de Porto Alegre (UFCSPA), Porto Alegre, Brazil; ^3^ Interuniversity Center for Healthy Aging, Talca, Chile; ^4^ Universidad de La Frontera, Facultad de Medicina, Departamento de Ciencias de la Rehabilitación, Temuco, Chile; ^5^ Universidad de La Frontera, Facultad de Ciencias Agropecuarias y Medioambiente, Doctorado en Ciencias Mención Biología Celular y Molecular Aplicada, Temuco, Chile; ^6^ Universidad de La Frontera, Facultad de Medicina, Departamento de Ciencias de Ciencias Básicas, Temuco, Chile; ^7^ Universidad de La Frontera, Facultad de Medicina, Doctorado en Ciencias Morfológicas, Temuco, Chile; ^8^ Universidad de La Frontera, Facultad de Medicina, Departamento de Ciencias Básicas, Centro de Biología Molecular y Farmacogenética, Temuco, Chile; ^9^ Department of Physical Therapy, Universidade Federal de Ciências da Saúde de Porto Alegre, Porto Alegre, Brazil; ^10^ Department of Physical Therapy, Georgia State University, Atlanta, GA, United States; ^11^ Universidad de La Frontera, Facultad de Medicina, Departamento de Medicina Interna, Temuco, Chile

**Keywords:** high intensity interval training, healthy aging, muscle strength, musculoskeletal system, muscle power

## Abstract

**Introduction:** Whether high-intensity interval training (HIIT) can improve lean mass, strength, and power of the lower limbs in young and older people is still under discussion. This study aimed to determine the effect of HIIT on lean mass, maximal strength, rate of force development (RFD), and muscle power of both lower limbs in healthy young and older adults. Secondarily, to compare the effects of HIIT between dominant vs. non-dominant lower limbs of each group.

**Materials and methods:** Healthy older (*n* = 9; 66 ± 6 years; BMI 27.1 ± 3.1 kg m^−2^) and young (*n* = 9; 21 ± 1 years; BMI 26.2 ± 2.8 kg m^−2^) men underwent 12 weeks of HIIT (3x/week) on a stationary bicycle. The evaluations were made before and after the HIIT program by dual energy X-ray absorptiometry (DEXA), anthropometry, force transducer and, Sit-to-Stand test. The outcomes analyzed were limb lean mass, thigh circumference, maximal voluntary isometric strength, RFD (Time intervals: 0–50, 50–100, 100–200, and 0–200 ms), and muscle power in both lower limbs.

**Results:** After 12 weeks of HIIT, non-dominant limb (NDL) showed increase in limb lean mass (*p* < 0.05) but without interaction (time*group). HIIT showed a gain in absolute maximal strength and also when adjusted for thigh circumference in the dominant lower limb (DL) in both groups. The RFD_0–200 ms_ showed differences between groups but without interaction. The RFD_0–50 ms_ of the NDL showed post-training improvements (*p* < 0.05) in both groups. Only the older group showed differences between DL vs. NDL in most of the RFD obtained post-intervention. In addition, post-HIIT muscle power gain was observed in both groups (*p* < 0.05), but mainly in older adults.

**Conclusion:** HIIT promotes increases in lean mass, maximal strength, early RFD, and lower limb muscle power in healthy older and young individuals. The differences shown between the DL and the NDL must be analyzed in future studies.

## 1 Introduction

Aging is associated with a progressive decrease in skeletal muscle mass, strength, and physical performance, a process defined as Sarcopenia by the European Working Group in Older People (EWGSOP 2) (Cruz-Jentoft et al., 2019). Worldwide, the prevalence of sarcopenia reaches ∼25% at 70 years of age and 40% over 80 years of age (Thompson, 2009). In Chile, according to the cut-off values of the European consensus, sarcopenia affects 19.1% (95% CI: 16.8%–21.8%) of people over 60 years of age (Lera L et al., 2017). Sarcopenia impacts the number, size, and type of muscle fibers, altering the quality and function of the muscular system (Venturelli et al., 2022). In addition, it has been closely associated with an increased risk of falls, loss of functionality, risk of hospitalization, and mortality in older people (Landi et al., 2012; Landi et al., 2013; Frontera, 2022).

Muscle power and rate of force development (RFD) are relevant neuromuscular parameters for physical performance in older people, and both are affected by the muscle remodeling process as a consequence of aging and sarcopenia (Varesco et al., 2019). Muscle power is understood as the ability to exert force rapidly (Reid and Fielding, 2012; Reid et al., 2014), and RFD is the speed at which the contractile elements of the skeletal muscle can develop force (Aagaard et al., 2002). These parameters play a fundamental role during tasks that demand explosive muscular actions of the lower limbs, such as getting up from a chair or climbing stairs (Guizelini et al., 2018; Hester et al., 2021). Both muscle power and RFD present an early and pronounced decrease during aging, even more, significant than muscle strength (Clark et al., 2013; Hester et al., 2021; Lomborg et al., 2022). In addition, both parameters specifically in lower limbs are significant predictors of the risk of falls, functional limitation, and disability in older people (Osawa et al., 2018; Bahat et al., 2021; Lomborget al., 2022). In this sense, preserving power and RFD is relevant for mobility and functional independence in this population.

Physical training is an intervention strategy that protects older people’s muscle strength and physical performance (Bao et al., 2020; Zhang et al., 2021). Although resistance exercise training is the most recommended type of training to maintain and increase skeletal muscle mass and strength condition (Dent et al., 2018; Beckwée et al., 2019), recent research has shown that power and explosive strength training are also associated with an improvement in functional capacity reducing the incidence of falls in frail and institutionalized older people (Izquierdo et al., 2021). The increase in RFD and power requires explosive movement training in a short time, which is possible to achieve during high-intensity interval training (HIIT). 

Muscular strength and power have shown a greater decline in knee extensor than in handgrip in older people (Samuel et al., 2012). Usually, the evaluation of muscle strength and power gain in strength training in older people is measured in the dominant/preferred limb of the participants (Müller et al., 2020) or in both limbs at the same time (T Balachandran et al., 2023). However, the presence of asymmetry of strength and muscular power in the lower limbs in older people in comparison with young subjects of 5%–10% is known (Carabello et al., 2010; LaRoche et al., 2017), which would affect performance in different functional tasks such as walking speed, climbing stairs, among other tasks (Portegijs et al., 2008; Straight et al., 2016). Furthermore, it has been reported that the performance of the preferred lower limb in young and adult subjects may differ from the contralateral limb (Carpes et al., 2010). Therefore, it seems important to analyze the effects of training involving bilateral mechanical stimuli on the demand of both lower limbs (i.e., bicycle) without assuming symmetry in older people. HIIT on a static bicycle is a training modality characterized by short, intermittent bursts of vigorous exercise alternated by rest periods or low-intensity recovery (Gibala et al., 2012).

HIIT has shown favorable effects on cardiorespiratory fitness in various health conditions and populations (Marriott et al., 2021; Wu et al., 2021). Studies have presented that HIIT is an excellent therapeutic alternative in young people for inducing neuromuscular adaptations such as muscle power and the ability to develop force rapidly during dynamic movements due to its short duration (∼20–30 min per session) and high-intensity work intervals (Martinez-Valdes et al., 2017; Schaun et al., 2019). In older people, recent studies propose that HIIT may be an effective training modality to improve the phenotypic characteristics of sarcopenia, these as the muscle function, muscle quantity, and physical performance (Hayes et al., 2021; Müller et al., 2021; Liu et al., 2022), as well as body composition, muscle strength, and muscle power (Sculthorpe et al., 2017; Buckinx et al., 2019; Alzar-Teruel et al., 2022). However, few studies have investigated the interaction between HIIT and lean mass, strength, rate of force development, and power in both lower limbs of healthy older and young people. Considering that HIIT can be a training alternative to maintain skeletal muscle function and preserve functional independence (Liu et al., 2022), this study aimed to determine the effect of HIIT on lean mass, maximal strength, rate of force development, and muscle power of both lower limb in healthy older and young people. Secondarily, to compare the effects of HIIT between dominant vs. non-dominant lower limbs of each group.

## 2 Materials and methods

### 2.1 Participants

Healthy older (*n* = 9; 66 ± 6 years; BMI 27.1 ± 3.1 kg m^-2^) and young (*n* = 9; 21 ± 1 years; BMI 26.2 ± 2.8 kg m^-2^) men were trained for 12 weeks with HIIT on a static bicycle. The present study is part of a larger project that determined the effects of HIIT on health parameters and quality of life among young *vs.* older people (Marzuca-Nassr et al., 2020; Artigas-Arias et al., 2021; Vidal-Seguel et al., 2023). The number of participants per group considered for this study was lower (*n* = 9) than the original project (*n* = 10), due to technical problems with the signals obtained from the force recording (criteria to determine RFD), so the participants with initial and final parameters complete data were included in the final statistical analysis.

### 2.2 Procedure

Men between 18–35 and 55–75 years old who were physically inactive (who did do not perform a programmed physical exercise more than twice a week) with body mass index (BMI) between 18.5 and 30 kg m^-2^ were recruited from May 2019 to September 2019. Participants with a history of surgery within the 3 months prior to selection, use of anticoagulants, cardiorespiratory or musculoskeletal conditions that have contraindications or limit the performance of exercise, diagnosis of type 2 diabetes mellitus (determined by fasting blood glucose >100 mg/dL or HbA1c values 6.5%), uncontrolled high blood pressure, use of nutritional supplements that can regulate skeletal muscle mass or cardiorespiratory fitness and smokers, were excluded. All the participants signed the informed consent after knowing the study’s aims, risks, and benefits. The study was approved by the Scientific Ethics Committee of the Universidad de La Frontera (Act No. 069_18, Folio 025_18, 2018) following the guidelines of the Declaration of Helsinki.

One week before the baseline assessments, participants completed a general health questionnaire to determine their eligibility for the study. Subsequently, each participant underwent the evaluations 48 h before the first HIIT session (PRE) and 48 h after the last training session (POST) at the University of La Frontera, Chile. Specifically, the participants performed a familiarization for evaluating of one repetition maximum (1RM) after the screening session. On a separate day, the participants arrived to the laboratory in a fasting state to perform the DEXA scan. After a standardized breakfast, anthropometric and physical evaluations were performed. Then, 12 h after, the VO_2max_ evaluation was performed (not considered in full for this report).

Anthropometric measurements were made to determine body composition (weight, height, Body Mass Index (BMI), thigh circumference, whole-body lean mass, and limb lean mass), strength (maximal strength and RFD of knee extensors), and muscle power of lower limbs. In addition, the dominance of the lower limbs was determined through a question: “If you had to kick a ball, with which lower limb would you do it more safely?” (van Melick et al., 2017). Additionally, dietary records and physical activity recorded together with the use of a pedometer were performed 3 days before starting HIIT and at week 11.

#### 2.2.1 Body composition

Each participant was placed barefoot on a stadiometer scale (SECA^®^ platform scale Madison, WI, United States) to measure body weight and height with 0.1 kg and 0.5 cm precisions, respectively. The BMI was determined with the weight (kg) and height squared (m^2^) ratio. The circumference of the thigh segment was determined with a SECA^®^ retractable tape measure graduated in (Madison, WI, United States), with the participant in a standing position, with both heels positioned on a drawn horizontal line and with a separation between feet of 15 cm.

Dual-energy X-ray absorptiometry (DEXA) scan determined total and lower limb lean mass (Lunar General Electric iDEXA, General Electric Medical Systems, Madison, WI, United States).

#### 2.2.2 Maximal strength and rate of force development

Maximal voluntary isometric strength was measured in both lower limbs (always maintaining the same order, first the dominant and then the non-dominant limbs). Before the strength measurement, the participants warm-up on a stationary bike for 5 min with a load 50 W and at 60 RPM. Then, each participant was placed on a chair with the trunk fully supported, with the hip and knee at 90°. A force transducer (Load Cell −500 lb. Sensortronics, United States) was used to measure knee extension’s maximal voluntary isometric strength. Before maximal strength recordings, participants performed four submaximal contractions at 50%, 70%, 80%, and 90% of their self-perceived maximum intensity for 3 s with 1 min of rest between each. The participants were instructed to perform a maximal isometric knee extension “as quickly as possible,” maintaining the requested movement for 3 s and repeating the test 3 times in both lower limbs. During the execution of each repetition, the participant was verbally motivated to achieve the best performance in the test. The maximum strength was obtained from the highest record of the three attempts.

**FIGURE 1:**
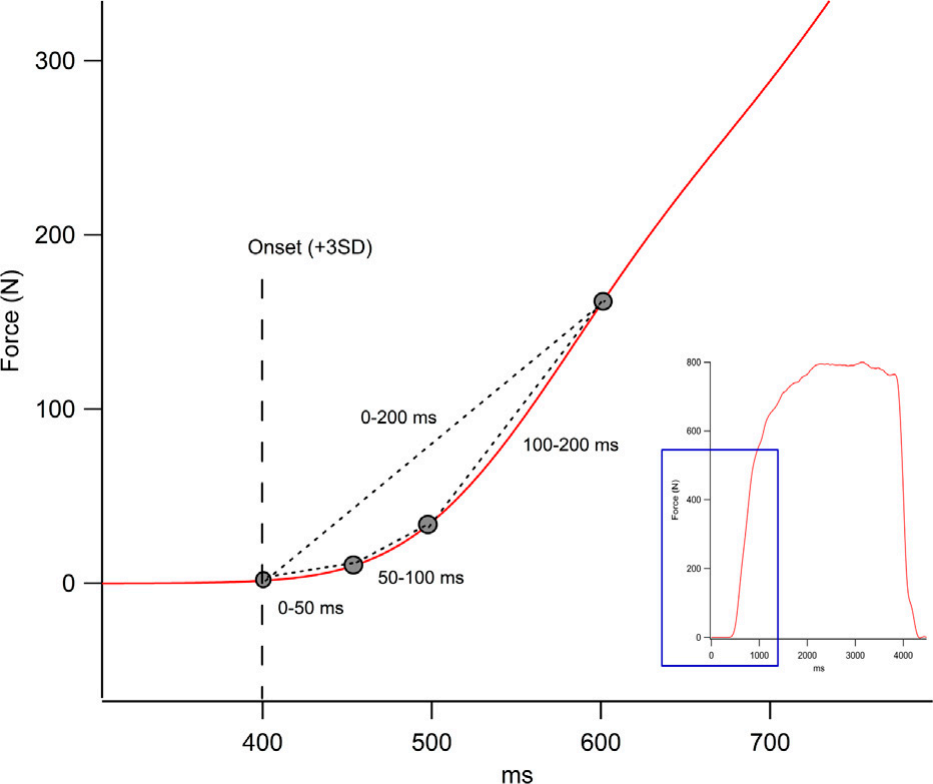
Representation of the raw force output onset in Newton (N) obtained during knee extension (thumbnail image on the right). The figure shows the rate of force development in the different intervals in milliseconds (ms).

The transducer captured the signal at 500 Hz with the IgorPro V6.1 software (WaveMetrics, Inc. United States), each signal was offset-corrected and smoothed with a 20 Hz low-pass filter, and each record’s maximum force was determined from a plateau of 2 s. The maximal voluntary isometric strength value of each repetition was used to normalize the rate of force development (RFD), defined as the relation between Force and Time (Maffiuletti et al., 2016). The onset of force was determined when it exceeded 3 SD of the rest signal before execution. The RFD was obtained in the first 200 ms of the force recording and was analyzed in 4 early time intervals of muscle contraction: 0–50 ms (RFD_0-50_), 50–100 ms (RFD_50-100_), 100–200 ms (RFD_100-200_) and 0–200 ms (RFD_0-200_) (Figure 1). The average value of the three repetitions was used to represent the RFD of each interval. The times of each interval were defined according to the recommendations of other studies to identify the initial muscle contraction during the development of rapid strength (Maffiuletti et al., 2016; Varesco et al., 2019).

Additionally, the maximal voluntary isometric strength obtained was adjusted concerning the lean mass (N/kg) and the thigh circumference (N/cm) in both lower limbs. Since the maximum knee extension strength is produced by mainly the quadriceps muscle, an gold standard measure (lower limb lean mass by DEXA scan) and a clinical measure (thigh circumference) that consider the amount of “quadriceps muscle mass” were considered to make strength adjustments.

#### 2.2.3 Sit to stand power

Five Times Sit to Stand test (STS) was conducted on an armless chair with a standardized height of 0.44 m from the floor to determine the muscle power of the lower limbs. The participant was seated with arms crossed at chest level. They were instructed to “stand up and sit down as fast as possible five times”. The participant was verbally encouraged during the timed test. Muscle power with STS was obtained through the equation proposed by Alcazar et al. (2018):

$$STS\;mean\;power=\frac{\left[Body\;mass\;x\;0.9\;x\;g\;x\;[Height\;x\;0.5-Chair\;height\right]}{Five\;STS\;time\;x\;0.1}$$

STS mean muscle power (W) is the product of STS mean speed and force. The average force is represented by the body mass displaced during the test (total body mass minus the mass of the legs and feet (0.90) multiplied by gravity (g = 9.8 m/s^2^), and the average velocity of the STS calculated as the vertical distance traveled by the center of mass (difference between the length of the legs (0.50 height) and the height of the chair) divided by the mean time taken to complete the STS test.

#### 2.2.4 High-intensity interval training (HIIT)

HIIT training was performed for 12 weeks, with a frequency of 3 times a week, on alternate days (Monday, Wednesday and Friday) on a stationary bike (Oxford^®^, BE2700) for both groups. The intervention was supervised by an experienced professional who continuously monitored each participant’s heart rate through a cardiac strap (Polar T31, Finland). To determine the intensity, the maximum aerobic capacity (VO_2max_) was used as a reference with continuous monitoring of the heart rate through the modified Astrand test (Marzuca-Nassr et al., 2020). Each session was performed at an intensity of 90% of the maximum heart rate obtained from the reference test, considering 1 min of exercise followed by 2 min of inactive rest, repeating each exercise/rest interval ten times. Heart rate measurements were taken at the start and end of each 1-min interval. Participants who completed at least 80% of their HIIT sessions were considered for the final analyses.

### 2.3 Statistical analysis

The normal distribution of all data was determined using the Shapiro-Wilk test. The baseline characteristics of the participants, such as age, weight, height, and BMI, among others, were compared between both groups using a *t*-test for independent samples. A two-way ANOVA was used with a between-subjects factor (older and young group) and a time factor (pre-HIIT and post-HIIT). If a main effect was found, a Bonferroni *post hoc* test was applied to determine the differences between pairs if necessary. Partial eta squared was used to estimate effect sizes and represented as *η* ^*2*^. Values of *η* ^*2*^ = 0.01 indicate a small effect, *η* ^*2*^ = 0.06 indicates a medium effect and *η* ^*2*^ = 0.14 indicates a large effect (Cohen, 1988). To determine the differences between dominant and non-dominant, an independent *t*-test was applied. All data are expressed as mean ± standard deviation (SD). All statistical analyzes were performed using SPSS V26.0 software (Chicago, Illinois, United States) and graphing were performed using GraphPad Prism V8.02 (GraphPad Software Inc. United States). The statistical significance level was set at *p* < 0.05.

## 3 Results

### 3.1 Baseline characteristics

Baseline characteristics of the participants are shown in Table 1. Both groups showed significant differences in age (*p* < 0.01). Older participants show lower baseline values of maximal oxygen consumption and higher time on the STS test (*p* < 0.01) than younger participants. Variables such as weight, height, BMI, whole-body lean mass, heart rate, and diastolic and systolic blood pressure did not show baseline differences between the two groups (*p* > 0.05). Dietary records were subjectively evaluated by a nutritionist, finding no intra-subject differences. The average daily steps also did not show intra-subject differences (*p* > 0.05). All participants completed 36 HIIT sessions (3 days a week for 12 weeks).

**TABLE 1:** Baseline characteristics of the participants.

	Young (n = 9)	Older (n = 9)	*p*-value
Age (years)	21 ± 1	66 ± 6	0.000*
Weight (kg)	77.7 ± 10.5	78.5 ± 12.3	0.894
Height (m)	1.7 ± 0.1	1.7 ± 0.1	0.372
BMI (kg/m^2^)	26.2 ± 2.8	27.1 ± 3.1	0.514
Heart Rate (b min^−1^)	70.8 ± 11.5	71.0 ± 10.7	0.983
DBP (mm Hg)	77.8 ± 6.4	80.6 ± 8.7	0.452
SBP (mmHg)	124.5 ± 4.7	128.4 ± 9.1	0.277
VO_2max_ (mL/kg/min)	30.0 ± 6.9	17.4 ± 4.0	0.000*
Whole body lean mass (kg)	55.5 ± 14.9	50.7 ± 6.6	0.394
Five Times STS Test (s)	7.0 ± 1.1	9.9 ± 2.0	0.002*

BMI, body mass index; DBP, diastolic blood pressure; SBP, systolic blood pressure; VO_2max_, maximal oxygen consumption; STS, Sit-To-Stand. Data are presented as mean and standard deviation. Symbol * denotes significant differences between groups at *p* < 0.05.

### 3.2 Body composition

The effects of 12 weeks of HIIT program on lower limb lean mass are presented in Table 2. No significant interaction between time and group in the dominant limb (DL: F_(1,16)=_0.98, *p* = 0.335) and non-dominant limb (NDL: F_(1,16)_ = 1.17, *p* = 0.294) was observed. However, a significant main effect of time was revealed (F_(1,16)_ = 7.61, *η* ^*2*^ = 0.322; *p* = 0.014) in the NDL with a relative increase of 2.2% ± 5.3% and 0.6% ± 4.11% in young and older, respectively.

**TABLE 2:** Body composition, maximum strength and power before and after HIIT, and the comparison between lower limbs.

Outcome	Young (n = 9)		Older (n = 9)		*Time/η^2^*	*Group/η^2^*	*Time x Group/η^2^*
	Pre	Post	Pre	Post			
**Dominant limb lean mass (kg)**	8.7 ± 0.9	8.9 ± 1.2 #	8.0 ± 1.1	8.0 ± 10.9	0.252/0.081	0.107/0.154	0.335/0.058
**Non-Dominant limb lean mass (kg)**	8.7 ± 0.9	8.9 ± 1.1	8.1 ± 1.0	8.2 ± 1.0	0.014/0.322*	0.163/0.118	0.294/0.068
**Dominant limb thigh circumference (cm)**	56.1 ± 3.6	55.4 ± 3.5	51.6 ± 3.6	52.1 ± 3.3	0.827/0.003	0.031/0.260*	0.146/0.127
**Non-Dominant limb thigh circumference (cm)**	55.8 ± 3.6	56.0 ± 3.4	50.9 ± 3.2	51.9 ± 4.0	0.158/0.121	0.013/0.328*	0.320/0.062
**Dominant limb MS (N)**	611.6 ± 177.8#	659.2 ± 169.8#	441.5 ± 108.7#	473.0 ± 132.2#	0.025/0.278*	0.020/0.296*	0.619/0.016
**Non-Dominant limb MS (N)**	450.7 ± 125.9	464.4 ± 125.9	312.9 ± 81.3	308.5 ± 99.3	0.601/0.017	0.011/0.341*	0.316/0.063
**Dominant limb MS/limb lean mass (N/kg)**	69.4 ± 17.2#	67.7 ± 15.3#	55.2 ± 12.3#	54.8 ± 11.8#	0.146/0.127	0.063/0.199	0.350/0.055
**Non-Dominant limb MS/limb lean mass (N/kg)**	52.0 ± 13.6	51.9 ± 12.3	39.2 ± 11.0	38.1 ± 12.3	0.647/0.013	0.033/0.259*	0.675/0.011
**Dominant limb MS/thigh circumference (N/cm)**	10.8 ± 2.8#	11.8 ± 2.8#	8.5 ± 1.9#	8.4 ± 1.9#	0.022/0.288*	0.039/0.240*	0.356/0.053
**Non-Dominant limb MS/thigh circumference (N/cm)**	8.0 ± 2.0	8.3 ± 2.0	6.1 ± 1.5	5.9 ± 1.8	0.958/0.000	0.029/0.263*	0.206/0.098

Abbreviations: MS, maximal strength; *η^2^*, Eta parcial; Data are presented as mean and standard deviation. Symbol * denotes main effect *p*-value < 0.05; Symbol # denotes differences between dominant and non-dominant lower limb *p*-value < 0.05.

On the other hand, thigh circumference did not show a significant interaction between time and group (DL: F_(1,16)_ = 2.32, *p* = 0.146; NDL: F_(1,16)_ = 1.05, *p* = 0.320, Table 2). However, a significant main effect of the group was revealed in the DL (F_(1,16)_ = 5.63, *η* ^*2*^ = 0.260, *p* = 0.03) and in the NDL (F_(1,16)_ = 7.79, *η* ^*2*^ = 0.328, *p* = 0.01), with a higher thigh circumference value in young participants but with a relative increase ∼1–2%.

### 3.3 Maximal strength

After HIIT training, muscle strength analysis showed no significant interaction effect between time and group for DL (F_(1,16)=_0.25, *p* = 0.619) and for NDL (F_(1,16)_ = 1.07, *p* = 0.316). A significant group main effect was revealed for DL (F_(1,16)_ = 6.71, *η* ^*2*^ = 0.296, *p* = 0.02) and NDL (F_(1,16)_ = 8.29, *η* ^*2*^ = 0.341, *p* = 0.01), with higher strength values in the young group before and after training. In addition, a significant main effect of time (F_(1,16)_ = 6.15, *η* ^*2*^ = 0.278; *p* = 0.025) in DL, showing a relative increase in young participants of 10.4% ± 16.0% and in older adults of 3.7% ± 9.3%, as shown in Table 2.

The muscle strength values adjusted for lean mass (Table 2), also showed no interaction effect between time and group in both DL and NDL (F_(1,16)_ = 0.92, *p* = 0.350; F_(1,16)_ = 0.18, *p* = 0.675, respectively). A significant group main effect was observed on NDL (F_(1,16)_ = 5.46, *η* ^*2*^ = 0.254, *p* = 0.03), showing differences between groups.

When adjusting the muscle strength to the thigh circumference (Table 2), there was no interaction effect between time and group in both DL and NDL (F_(1,16)_ = 0.90, *p* = 0.356; F (1,16) = 1.72, *p* = 0.207, respectively). However, a significant group main effect was revealed on the DL (F_(1,16)_ = 5.06, *η* ^*2*^ = 0.240, *p* = 0.03) and on the NDL (F_(1,16)_ = 5.71, *η* ^*2*^ = 0.263, *p* = 0.02), showing a higher strength value in the young group before and after training. In addition, a significant main effect of time (F_(1,16)_ = 6.48, *η* ^*2*^ = 0.288; *p* = 0.022) in the DL that showed a relative increase of 11.7% ± 17.4% in the young group and 6.21% ± 15.1% in the older adult group.

### 3.4 Rate of force development

**FIGURE 2:**
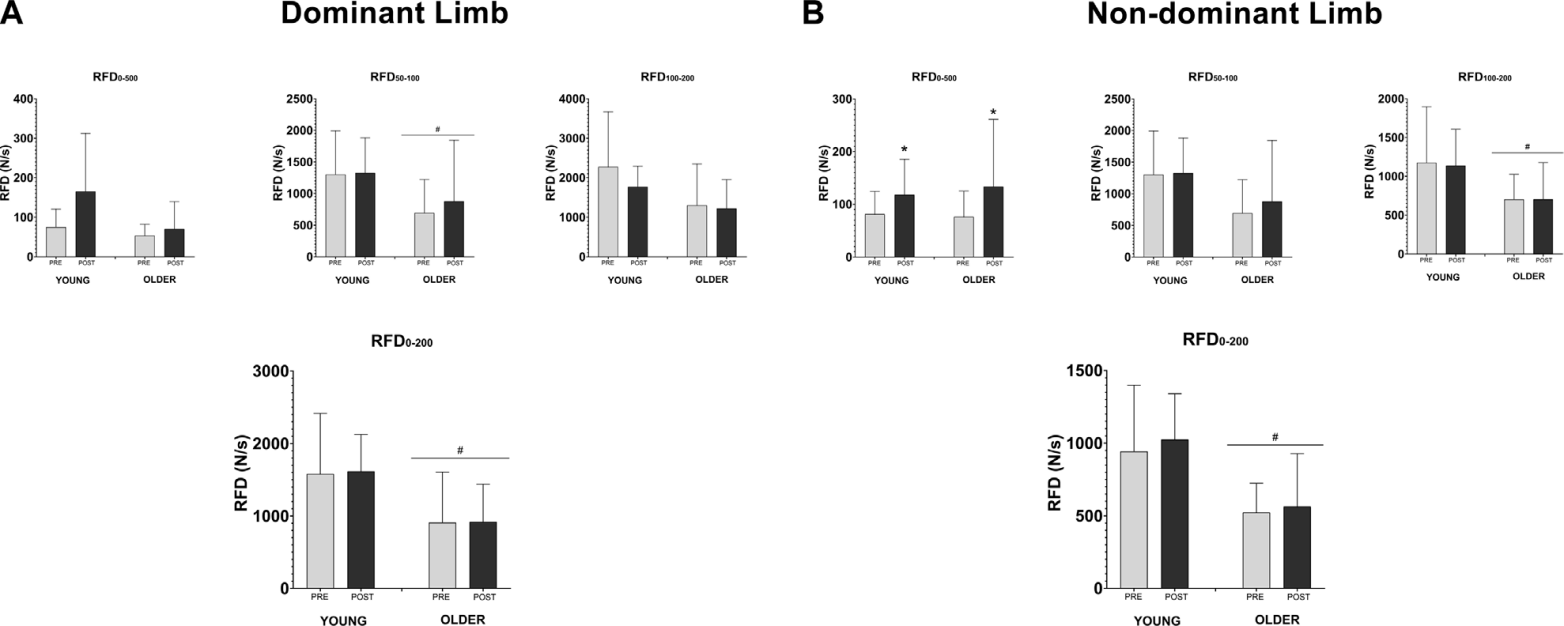
Effects of HIIT training on rate of force development (RFD). Each time interval was expressed on the graph (0–50, 50–100, 100–200, and 0–200 ms). Panel A shows the values of the dominant lower limb at each RFD interval. Panel B shows the values of the non-dominant lower limb in each RFD interval. The graphs are presented in mean and standard deviation. The symbol * denotes time effect with *p* < 0.05, and # denotes group effect with *p* < 0.05.

In the analysis of the RFD after 12 weeks of HIIT program, no significant interaction effect between time and group was found in both DL and NDL in all four-time intervals analyzed (RFD_0–50 ms,_ RFD_50–100 ms,_ RFD_100–200 ms,_ RFD_0–200 ms_, all *p* > 0.05). A significant group main effect on DL was revealed at RFD_0–50 ms_ interval (F_(1,16)_ = 8.45, *η* ^*2*^ = 0.346, *p* = 0.01) and RFD_0–200 ms_ interval (F_(1,16)_ = 6.46, *η* ^*2*^ = 0.288, *p* = 0.02) (Figure 2A). Furthermore, in the NDL was observed also a significant group effect on DL for RFD_100–200 ms_ interval (F_(1,16)_ = 5.50, *η* ^*2*^ = 0.256, *p* = 0.03) and RFD_0–200 ms_ interval (F_(1,16)_ = 11.43, *η* ^*2*^ = 0.417, *p* = 0.004) (Figure 2B).

In addition, a significant main effect of time was evidenced in RFD_0–50 ms_ in the NDL (F_(1,16)_ = 6.55, *η* ^*2*^ = 0.291; *p* = 0.021), where a relative increase of 105% ± 225.3% was observed in the young group and 52.1% ± 61.0% in the group of older adults (Figure 2B).

### 3.5 Muscle power

**FIGURE 3:**
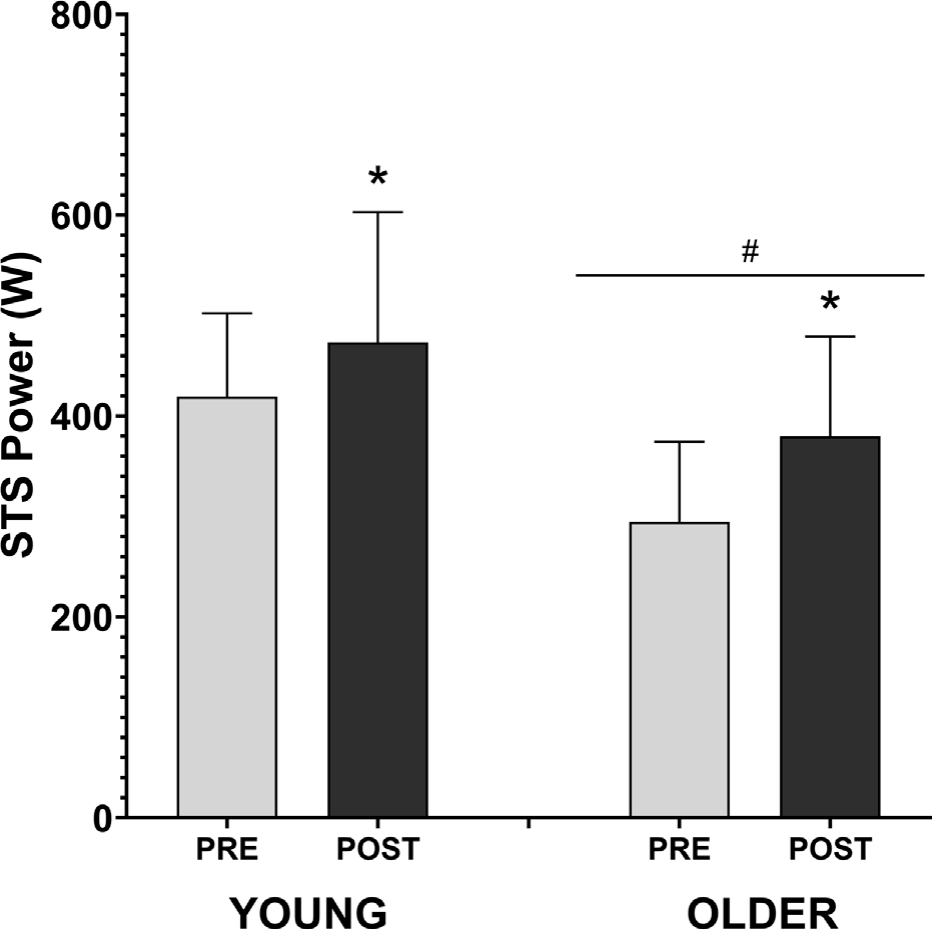
Effects of HIIT training on sit to stand power muscle. The graphs are presented in mean and standard deviation. The symbol * denotes time effect with *p* < 0.05, and # denotes group effect with *p* < 0.05.

HIIT training produced higher increase on STS power in young participants (from 419.5 ± 82.7 to 473.4 ± 129.4 W) when compared to older people (from 294.8 ± 79.5 to 380.1 ± 99.0 W) (Time effect: F_(1,16)_ = 13.13, *η* ^*2*^ = 0.451; *p* = 0.002). A relative increase of 12.1% ± 16.8% was observed in the young group and 34.7% ± 39.0% in the older group. A significant main effect was obtained between the young and older groups (F_(1,16)_ = 6.46, *η* ^*2*^ = 0.288, *p* = 0.02), but with no time*group interaction effect (F (1,16) = 0.67, *p* = 0.425) (Figure 3).

### 3.6 Dominant vs. non-dominant lower limbs

When comparing dominant and non-dominant limbs (Table 2), lean mass and thigh circumference did not show differences in either group (*p* > 0.05, both). On the other hand, muscle strength showed higher records in DL over NDL, before and after the HIIT program (*p* < 0.05) in both groups. Likewise, the strength adjusted for both lean mass and thigh circumference showed the same differences as the absolute values of maximum strength (*p* < 0.05).

When comparing the RFD between dominant and non-dominant limbs, there are differences only in the older group in the intervals RFD_50–100 ms_, RFD_100–200 ms_ and RFD_0–200 ms_ (*p* < 0.05) after the HIIT program.

## 4 Discussion

This study aimed to determine the effect of HIIT on lean mass, maximal strength, rate of force development (RFD), and lower limb muscle power in healthy young and older participants. The results show that HIIT increased lean mass and maximum strength in both groups but with differences between the dominant and non-dominant lower limbs. In the case of RFD, an increase in the early time interval of force (RFD_0–50 ms_) was only observed in the non-dominant lower limb in both groups but not in the other RFD intervals. When comparing the RFD between dominant and non-dominant members, there are differences only in the older age group after the HIIT program. Finally, the muscular power obtained with the STS test showed an increase in post-HIIT values in both groups, with a greater relative increase in the older group. However, the results showed no time*group interaction in response to HIIT training.

HIIT has been shown to be a safe physical training modality in older people, with beneficial effects on total muscle volume and maximum strength (Moro et al., 2017; Callahan et al., 2021). Consistent with this, our data show those 12 weeks of HIIT successfully increase lean mass and maximum strength in healthy younger and older individuals. Furthermore, these parameters have been strongly related to decreased risk of falls, hospitalizations, and morbidity and mortality in older people (Mitchell et al., 2012; Alajlouni et al., 2023). Strength measurement, specifically of knee extensors, has been suggested as a way of early identification of strength reduction in aging (Abdalla et al., 2020). However, muscle strength is usually adjusted only for body weight, without considering the characteristics of size or composition of the analyzed member (Jaric et al., 2002; Machado et al., 2022). Our results show that HIIT promotes improvements in the absolute maximum strength of the knee extensors. When the obtained strength is adjusted for thigh circumference, the results are similar to the absolute values, but when adjusted for legs lean mass, they change. Since the relationship between strength and sarcopenia is not linear, training sessions that may influence body composition or segmental anthropometric parameters, such as lower limb HIIT, should be analyzed together with muscle strength gains to avoid bias in its interpretation (Compston et al., 2014; Abdalla et al., 2020).

Muscle power is a crucial factor in the functional capacity of older people, related to gait speed and risk of falls (Bean et al., 2010; Reid and Fielding, 2012). As previously mentioned, muscle power declines earlier than skeletal muscle mass and strength with aging. For example, Goodpaster et al. (2006), studied older men and women who exhibited a loss of strength three times greater than the decrease in muscle mass over 3 years of follow-up. Then, in the study by Bahat et al. (2021), they found that lower muscle power was more prevalent than lower muscle strength in older adults. Furthermore, Wiegmann et al. (2021), observed a higher and earlier RFD loss than strength and muscle mass over 6 years of follow-up. Our results showed that a HIIT program increases muscle power (12.1% ± 16.8% in the young group and 34.7% ± 39.0% in the older group) and RFD_0–50 ms_ (105% ± 225.3% in the group young and 52.1% ± 61.0% in the group of older adults). Few studies have looked at the effects of HIIT on muscle function such as muscle power in older people. Müller et al. (2020), demonstrated an increase in RFD after 16 weeks of HIIT when combined with resistance or power training in older people. Speed of muscle recruitment and the intensity of the training seems to be determining factors for the rapid gain in strength and would depend on neuromuscular factors (Gerstner et al., 2017), which could explain the improvements in the initial strength intervals in older people. Sarcopenia in older people presents skeletal muscle atrophy, functional deficit and, slow muscle response. With its intense and fast exercise, HIIT could promote the preservation of skeletal muscle mass, strength, and power (Padilla Colon et al., 2014; Coletti et al., 2022).

The muscle qualities of both lower limbs after HIIT program have been rarely reported, particularly in older people (Hurst et al., 2019). The difference in strength (asymmetry) between knee extensors in older subjects (15%–20%) is greater than in younger individuals (∼10%) (Perry et al., 2007; Carabello et al., 2010). Some studies have associated a greater asymmetry in the strength of the lower limbs with a greater risk of falls and functional limitations in older people (Straight et al., 2016; LaRoche et al., 2017). In our study, we analyzed both lower limbs due to the characteristics of the bilateral stimulus that HIIT program presents when performed on a bicycle, which would therefore demand both lower limbs in a similar way. However, we observed differences between DL in relation to NDL in strength and RFD at baseline in both groups and that were maintained post-training, accentuating in older people. Carpes et al. (2010), observed than in subjects trained on cycling, the performance of the preferred lower limb may differ from the contralateral one, which could explain why the differences between both limbs were maintained with this training and facilitate the increase in asymmetry observed in the older people. Nunes et al. (2022), reported that traditional lower limb strength training (with bilateral strength exercises) in older women improved limb asymmetry, but not all responded in the same way. Therefore, as some authors indicate, the baseline asymmetries between the lower limbs could cause asymmetric strength gains, mainly due to the type of exercise used that allows more or less force to be applied with one limb compared to the other (Nunes et al., 2022). Clinically, it seems relevant to consider asymmetry in the older people in evaluations and training, since it would allow us to better estimate the performance of the lower limbs and a better understanding of the gains and adaptations that muscle function achieves in this population (LaRoche et al., 2017).

This study has limitations. The small sample size may limit some scope of the results. However, the analysis of both dominant/non-dominant lower limbs can broaden the discussion possibilities. In this sense, the lack of randomization in the order of the strength tests did not allow us to analyze whether the strength gains could have been influenced by an order evaluation effect, which should be considered for future experiments, despite the fact that recent studies recommend performing a pre-assessment familiarization beginning with the dominant lower limb (as performed in this study) (Chan et al., 2020). Future studies should consider larger samples of older people who have evaluated the muscle strength and power of both lower limbs to understand the contributions of HIIT in this regard. On the other hand, muscle power analysis often focuses on the knee extensor muscles. It would be essential to evaluate how HIIT promotes favorable effects in the other muscle groups associated with the stability of the lower limbs.

In conclusion, HIIT promotes increases in lean mass, strength, early RFD, and lower limb muscle power in healthy old and young individuals. The differences shown between the DL and the NDL must be analyzed in future studies. The real scope of skeletal muscle adaptation in HIIT must be addressed in more complex studies of muscle function.

## Data Availability

The raw data supporting the conclusions of this article will be made available by the authors, upon further evaluation by contacting the corresponding author.
